# Clozapine and paliperidone palmitate antipsychotic combination in treatment-resistant schizophrenia and other psychotic disorders: A retrospective 6-month mirror-image study

**DOI:** 10.1192/j.eurpsy.2020.72

**Published:** 2020-07-16

**Authors:** Miquel Bioque, Eduard Parellada, Clemente García-Rizo, Sílvia Amoretti, Adriana Fortea, Giovanni Oriolo, Pol Palau, Ester Boix-Quintana, Gemma Safont, Miquel Bernardo

**Affiliations:** 1 Barcelona Clínic Schizophrenia Unit (BCSU), Neuroscience Institute, Hospital Clínic de Barcelona, Barcelona, Spain; 2 Institut d’Investigacions Biomèdiques August Pi i Sunyer (IDIBAPS), Barcelona, Spain; 3 Centro de Investigación Biomédica en red en salud Mental (CIBERSAM), Madrid, Spain; 4 Department of Medicine, University of Barcelona, Barcelona, Spain; 5 Child and Adolescent Psychiatry and Psychology Department, Institute of Neurosciences, Hospital Clínic de Barcelona, University of Barcelona, Barcelona, Spain; 6 Fundació Clinic per a la Recerca Biomèdica (FCRB), Barcelona, Spain; 7 Psychiatry Department, Neuroscience Institute, Hospital Clínic de Barcelona, University of Barcelona, Barcelona, Spain; 8 Day Hospital, Centre Psicoteràpia Barcelona (CPB), Barcelona, Spain; 9 Psychiatry Department, Hospital General de Granollers, Granollers, Spain; 10 Psychiatry Department, Hospital de Mataró, Mataró, Spain; 11 Psychiatry Department, Hospital Universitari Mútua de Terrassa, Terrassa, Spain; 12 Department of Medicine, University of Barcelona, Barcelona, Spain

**Keywords:** Clozapine, long-acting injectable antipsychotics, paliperidone palmitate, psychosis, treatment resistant schizophrenia.

## Abstract

**Background:**

Around 30% of patients with schizophrenia are considered treatment resistant (TRS). Only around 40% of TRS patients respond to clozapine. Long acting injectable antipsychotics could be a useful augmentation strategy for nonresponders.

**Methods:**

We conducted a multicenter, observational, naturalistic, retrospective, 6-month mirror-image study to evaluate the efficacy and tolerability of clozapine and paliperidone palmitate association in 50 patients with TRS and other psychotic disorders. Clinical outcomes and side effects were systematically assessed.

**Results:**

Six months after starting the combined treatment, participants showed a significant relief of symptoms, decreasing the Brief Psychiatric Rating Scale total score from 18.32 ± 7.71 to 7.84 ± 5.16 (*p* < 0.001). The number of hospitalizations, the length of hospital stays and the number of visits to emergency services also decreased, while an increase of the functionality was observed (Personal and Social Performance total score increased from 46.06 ± 118.7 to 60.86 ± 18.68, *p* < 0.001). There was also a significant decrease in the number and severity of side effects with the combination therapy, decreasing the Udvalg for Kliniske Undersogelser total score from 10.76 ± 8.04 to 8.82 ± 6.63 (*p* = 0.004).

**Conclusions:**

This study provides the first evidence that combining clozapine with paliperidone palmitate in patients with TRS and other psychotic disorders could be effective and safe, suggesting further research with randomized controlled trials of augmentation strategies for clozapine nonresponder patients.

**Policy Significance Statement:**

Patients with psychotic disorders such as schizophrenia show a variable response to antipsychotic treatments. Around 30% of patients are considered treatment resistant, indicated by insufficient symptom control to at least two different drugs. In these resistant cases, clozapine should be indicated, as it has shown to be superior to other options. However, only 40% of patients respond to clozapine, being necessary to establish which treatments could best potentiate clozapine action. Combining clozapine with long acting injectable antipsychotics, and particularly paliperidone palmitate, could be a useful strategy. We conducted a multicenter study of 50 patients with treatment-resistant schizophrenia and other psychotic disorders comparing the efficacy and tolerability in the 6 month-period prior and after starting the clozapine and paliperidone palmitate association. Our study suggests that this combination could be effective and safer, laying the groundwork for future clinical trials with this combination.

## Introduction

Pharmacological response to antipsychotic treatment for patients with chronic psychotic disorders is heterogeneous, being mediated by two principal barriers: treatment resistance and treatment nonadherence [1–3].

Recommendations after an inadequate first antipsychotic response include waiting for a delayed response, dose adjustment and switching to a second antipsychotic [4,5]. Around 30% of patients with schizophrenia are considered treatment resistant (TRS), indicated by insufficient symptom control and low response rates to at least two adequate antipsychotic trials [4,6–9]. In case of treatment resistance, clozapine should be indicated, as it has shown to be superior to other antipsychotics [10–12].

Besides, both chronic and first-episode patients with schizophrenia show high treatment discontinuation rates [11,13]. Multi-episodic courses linked to relapses may lead to poor outcomes and negative effects on brain integrity, facilitating conditions that seriously interfere with rehabilitation efforts [14,15]. Long-acting injectable (LAI) antipsychotics were developed with intention to improve schizophrenia patients’ long-term outcomes by reducing the high rates of relapses and rehospitalizations due to treatment discontinuation [16]. Their use has been associated to a 30% decrease in mortality risk [17]. In concrete, paliperidone palmitate, either in 1-month or three-month formulation, have shown to be effective to reduce the number of relapses related to treatment lack of adherence, both in the short- and in the long term [2,18–21]. However, despite LAI antipsychotics have documented their value for preventing relapses, they are significantly underused due to different barriers [16,22–26]. Interestingly Treatment Response and Resistance in Psychosis (TRRIP) Working Group Consensus recommends the use of LAI antipsychotics during at least 4 months before initiation clozapine in order to differentiate treatment resistant-psychosis from resistance to be treated [6].

Clozapine monotherapy is considered by most guidelines and expert consensus the most effective medication for TRS [27–30]. However, only 40% of people will meet response criteria, being necessary to establish well evidenced-bases clozapine augmentation strategies [31–33]. Recent systematic meta-review of available evidence and international expert consensus point that augmentation with second-generation antipsychotics and first-generation antipsychotics can be beneficial, but the supporting evidence is from low-quality studies [32,33]. A previous retrospective 1-year mirror-image study reported that clozapine and various LAI antipsychotics (mainly first generation and risperidone) combination was beneficial and safe in a cohort of 17 multi-episode patients diagnosed with schizophrenia and schizoaffective disorder [3]. This previous study did not include paliperidone palmitate augmentation, which could a priori be a suitable drug to enhance the effect of clozapine as paliperidone presents a different receptor profile. Besides, in a previous pilot data collection of patients combining clozapine with oral, extended-release paliperidone, or paliperidone palmitate, we had found interesting outcomes that need to be studied more deeply [34]. Moreover, as other polytherapy regimens, this combination was already being used in the clinical practice of the participating centers, so we identified the need to study whether this combination was effective and safe.

The aim of this study was to evaluate the efficacy and tolerability of clozapine and paliperidone palmitate association in patients with TRS and other psychotic disorders.

## Subjects and Methods

### Subjects

**Figure 1.**
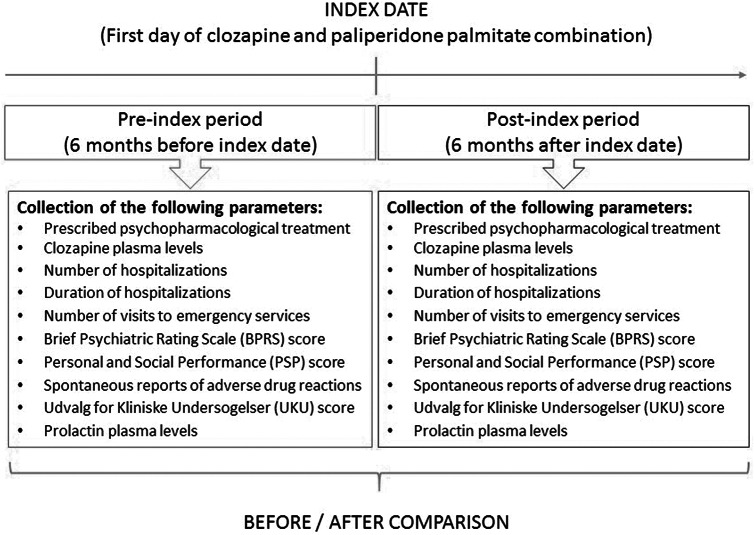
Study Protocol.

We conducted a multicenter, observational, naturalistic, retrospective 6-month mirror-image study to assess efficacy and tolerability of the clozapine and paliperidone palmitate combination in patients with a treatment-resistant psychotic disorder by comparing data relative to patients 6 months before and after initiating this combination. The index date was defined as the starting date of the clozapine and paliperidone palmitate combination (see Figure 1). The duration of 6 months was prespecified, considering that paliperidone palmitate steady state may not be reached until 4–5 months after commencing treatment [35] and that other similar mirror-image studies involving LAI antipsychotics also covered 6 months before and after initiating the studied strategy [36].

This retrospective observational study was conducted from January 2017 to April 2019 in inpatients or outpatients’ services of four public hospitals: Hospital Clínic de Barcelona, Hospital General de Granollers, Hospital de Mataró, and Hospital Universitari Mútua de Terrassa. Because this was an observational retrospective study, patients were not required to provide informed consent. The present study was approved and authorized by the respective departments of psychiatry in which it was conducted and performed in accordance with the ethical standards laid down in the 1964 Declaration of Helsinki and its later amendments.

Inclusion criteria were: (a) patients being followed-up in one of the four participating centers during the recruiting period; (b) patients ≥18 years of age and diagnosed of schizophrenia, schizoaffective disorder, delusional disorder, or bipolar disorder with psychotic features according to The Diagnostic and Statistical Manual of Mental Disorders, Fifth Edition (DSM-5) criteria [37]; (c) patients that, according to the referring psychiatrist, could take benefit of a change from the previous treatment (due to lack of efficacy, poor adherence, side effects, or other reasons) after having used under adequate dose of at least two different antipsychotics during 6–8 weeks with at least one of them being a nonclozapine atypical antipsychotic.

### Psychopharmacological treatment

Being a naturalistic study, there were no specific guidelines for treatments, so patients received the combination antipsychotic treatment based on the clinician’s choice. Dosing, co-medications, or treatment changes were based on clinical necessity. Clozapine mean daily dose, plasma mean levels when available, paliperidone palmitate formulation (monthly or quarterly), paliperidone palmitate mean monthly dose, treatment starting dates, and coadjuvant antipsychotic, antidepressant, anxiolytic, mood stabilizer, anticholinergic, or other psychopharmacological treatments were recorded. Being a retrospective data collection, patients included did not lead a different patient care that deviated from the regular treatment. In participants with other diagnosis than schizophrenia, clozapine represented an off-label treatment. This use was specified in each participant’s medical file.

### Clinical and tolerability assessment

Sociodemographic, clinical, and treatment data were collected using a unique, homogenized electronic data notebook. Clinical data regarding pre- and post-switch period were recorded, including the number and length of hospitalizations and the number of visits to emergency services. Main clinical outcomes were assessed by means of the Bech’s version of the Brief Psychiatric Rating Scale (BPRS) [38,39], which was applied by trained raters in each of the two visits to evaluate the psychopathological state of the patients. The Bech’s version of the BPRS assesses the level of 18 symptom constructs to measure psychopathology severity, where each symptom is rated 0–4 from absence to most severe [39].

In order to identify potential predictors of efficacy and following Leucht et al. recommendations [40], we classified the sample in five groups according to changes in BPRS total scores between index date and the 6-month evaluation: participants with an increase in the BPRS score (meaning clinical worsening) and participants with <25%, 25–49%, 50–74% or >75% reduction, respectively. In order to avoid multiple testing [40], we analyzed potential predictors associated with a reduction of ≥25% of the BPRS score, considering that this decrease could be associated with significant clinical improvements in treatment-resistant patients. Thus, participants with a BPRS reduction of 25% or more were considered “responders,” while those with lower reductions or increases were considered “nonresponders.”

Other recorded clinical data included Personal and Social Performance (PSP) score [41]. The PSP scale was developed to evaluate social and personal functioning and considers four areas (socially useful activities; personal and social relationships; self-care; and disturbing and aggressive behaviors). For filling it, the clinician must assign an initial six-degree of severity to each area (from absent over mild, manifest, marked, severe to very severe difficulties in the given area). The final result is a single measurement from 0 to 100% where higher scores reflect better personal and social functioning.

In order to assess in detail the adverse drug reactions, two procedures were followed: (a) spontaneous reports of ADRs Adverse Drug Reaction and (b) systematic assessment of the effects targeted in the Scale of the Udvalg for Kliniske Undersogelser (UKU) [42], a comprehensive rating scale designed to assess general side effects of psychotropic drugs. Investigators also reported any specific treatment or change in the prescription due to ADRs appearance, including antipsychotic discontinuation, dose reduction or start of an anticholinergic drug. Those ADRs that made the clinician change the usual practice by, for example, requesting a blood test that was not previously scheduled, sending the patient to the emergency room or hospitalizing them was considered as serious.

### Statistical analysis

Comparisons of clinical and treatment variables between the pre- and post-switch period were performed. We have a priori determined that a sample size of 34 patients was required to detect a difference of 0.5 (± 1 standard deviation) in the mean number of psychiatric hospital admissions and a difference of 5 (± 10 standard deviations) in the mean number of bed days with a power of 0.80 and a two-sided 0.05 significance level. Baseline characteristics were summarized using descriptive statistics. Means and standard deviations (SD) for continuous variables and counts and percentages for categorical variables were calculated. Differences were tested for statistical significance using Wilcoxon rank tests for non-normally distributed variables, confirmed by the Kolmogorov–Smirnov test. A McNemar’s test was used to asses significant changes in proportions between the two periods observed. In each analysis, a pairwise exclusion of missing was used.

We analyzed a set of potential predictors of response related to the pre-index period through different tests. For categorical variables (taking/not taking clozapine, taking/not taking paliperidone palmitate, taking/not taking oral paliperidone or risperidone, being under antipsychotic monotherapy vs polytherapy and diagnosis) we used a Chi-square test for independence (with Yates Continuity Correction). For continuous variables (age at index date, age at disorder onset, duration of disorder), we used a Mann–Whitney U test.

A value of *p* < 0.05 was taken to be statistically significant in all analysis. Data were managed and analyzed with the IBM SPSS Statistics v.23.

The present study was reported following the Strengthening the Reporting of Observational Studies in Epidemiology (STROBE) statement (for STROBE checklist, see Supplementary Material).

## Results

Demographic, clinical, and treatment characteristics of the 50 participants in this study are shown in Table 1.

**Table 1.** Baseline sociodemographic, clinical and treatment characteristics of the sample.

	(*n* = 50)
Gender—no. (%)	
Female	13 (26%)
Male	37 (74%)
Age—years (mean [SD])	40.82 (11.78)
Age at disorder onset—years (mean [SD])	27.30 (8.23)
Duration of illness—years (mean [SD])	13.52 (9.74)
Diagnosis—no. (%)	
Schizophrenia	33 (66%)
Schizoaffective disorder	13 (26%)
Delusional disorder	2 (4%)
Bipolar disorder	2 (4%)
Main reasons for initiating combined treatment—no. (%)	
Lack of efficacy	25 (50%)
Poor adherence	9 (18%)
Both	7 (14%)
Other reasons	9 (18%)

Abbreviation: SD, standard deviation.

Participants maintained the treatment combination for a mean of 5.24 months (±1.39). Thirty-seven patients (74%) remained with the combined treatment all the 6 months of observation period after associating treatments. See study flow chart in Supplementary Material.

Table 2 shows a comparison of psychopharmacological treatment between the 6 months preceding and the 6 months following clozapine and paliperidone palmitate combination. During the 6 months before the association treatment, 32 subjects (64%) were taking clozapine, 13 (26%) were taking paliperidone palmitate, and 5 (10%) did not take either of the two in the previous period.

**Table 2.** Comparison of psychopharmacological treatment between the 6 months preceding and the 6 months following clozapine and paliperidone palmitate combination.

	6 months before	6 months after combination treatment	Statistic	*p* value
Subjects taking clozapine	32/50 (64%)	50/50 (100%)		
Mean daily dose—mg/day (SD)	330.47 (189.52)	265.50 (164.82)	*Z* = −1.86	*p* = 0.062
Subjects taking paliperidone palmitate	13/50 (24%)	50/50 (100%)		
Mean monthly dose—mg/month (SD)	134.38 (34)	123 (33.82)	*Z* = −0.68	*p* = 0.49
Subjects with other psychopharmacological treatments				
Antipsychotics	40/50 (80%)	9/50 (18%)	–	*p* < 0.001*
Anticholinergics	10/50 (20%)	6/50 (12%)	–	0.22
Antidepressants	10/50 (20%)	13/50 (26%)	–	0.55
Mood stabilizers	14/50 (28%)	11/50 (22%)	–	0.25
Benzodiazepines	26/50 (52%)	9/50 (18%)	–	*p* < 0.001*

* Significant value *p* < 0.05

During the 6 months after the association treatment, 3 of the 50 patients (6%) switched to the equivalent 3-month formulation of paliperidone palmitate; the remaining patients were kept in the monthly formulation.

The use of other antipsychotics decreased from 80% of the participants in the precombination period to 18% during the 6 months postcombination period (*p* < 0.001). The most common antipsychotics used during the precombination period were risperidone (*n* = 14), haloperidol (*n* = 7), oral paliperidone (*n* = 7), and aripiprazole (*n* = 7). Five participants switched from other LAI antipsychotics to paliperidone palmitate (three from risperidone LAI and two from zuclopenthixol decanoate). There was also a significant decrease in the proportion of participants taking benzodiazepines (52% vs. 18%, *p* < 0.001), but not in the use of anticholinergic drugs, antidepressants or mood stabilizers.

Main efficacy outcomes are presented in Table 3. In the population studied, the combination of clozapine and paliperidone palmitate showed a significant decrease in the number and length of hospitalizations, number of visits to emergency services and in the BPRS total score.

**Table 3.** Comparison of clinical results between the 6 months preceding and the 6 months following clozapine and paliperidone palmitate combination.

	6 months before combination treatment	6 months after combination treatment	Statistic	*p* value
Mean number of hospitalizations (SD)	0.86 (0.7)	0.36 (0.63)	*Z* = -4.11	*p* < 0.001*
Mean total duration of hospitalizations (days; SD)	39.08 (53.61)	18.26 (41.23)	*Z* = −3.02	*p* = 0.002*
Mean number of visits to emergency services (SD)	1.26 (1.7)	0.46 (0.97)	*Z* = −3.57	*p* < 0.001*
BPRS total score (mean; SD)	18.32 (7.71)	7.84 (5.16)	*Z* = -5.99	*p* < 0.001*
Participants changes in BPRS total score				
Increase—*n* (%)	–	2 (4)	–	–
<25% reduction *n* (%)	–	4 (8)	–	–
25–-49% reduction *n* (%)	–	7 (14)	–	–
50–-74% reduction *n* (%)	–	28 (46)	–	–
>75% reduction *n* (%)	–	9 (18)	–	–
PSP total score (mean; SD)	46.06 (18.7)	60.86 (18.68)	*Z* = −5.34	*p* < 0.001*
Self-care	1.54 (1.2)	0.9 (0.95)	*Z* = −4.32	*p* < 0.001*
Personal and social relationships	2.68 (0.91)	1.88 (1.02)	*Z* = −4.72	*p* < 0.001*
Socially useful activities	3.06 (1.15)	2.08 (1.27)	*Z* = −5.07	*p* < 0.001*
Disturbing and aggressive behavior	1.14 (1.37)	0.28 (0.67)	*Z* = −4.27	*p* < 0.001*

Abbreviations: BPRS: Brief Psychiatric Rating Scale; PSP: Personal and Social Performance scale; SD, standard deviation.

* Significant value *p* < 0.05.

None of the potential predictors of response studied reached statistical significance. Age at index-date showed a statistical tendency (*U* = 70.5; *p* = 0.066), meaning that the group of responders showed a nonsignificant tendency to be younger than nonresponders (mean age at index-date: 24.1 vs. 35.75 years old).

A significant increase in the PSP total score was also detected, with significant improvements in all sub-domains.

Finally, data regarding the combined treatment tolerance is shown in Table 4. There was a significant decrease in the number and severity of side effects with the combination therapy. The decrease of the UKU total score was related to the psychiatric and neurological domains. There was a nonsignificant elevation of mean prolactin plasma levels. There was no evidence of an increased risk of agranulocytosis with the combination therapy.

**Table 4.** Comparison of tolerability results between the 6 months preceding and the 6 months following clozapine and paliperidone palmitate combination.

	6 months before combination treatment	6 months after combination treatment	Statistic	*p* value
UKU total score (*n* = 50)	10.76 (8.04)	8.82 (6.63)	*Z* = −2.88	*p* = 0.004*
UKU psychic side effects	5.48 (4.13)	3.92 (3.17)	*Z* = −2.84	*p* = 0.004*
UKU neurologic side effects	1.18 (1.67)	0.74 (1.08)	*Z* = −2.12	*p* = 0.034*
UKU autonomic side effects	1.64 (2.16)	1.7 (2.02)	*Z* = −0.56	*p* = 0.96
UKU other side effects	2.46 (2.72)	2.46 (2.72)	*Z* = 0	*p* = 1
Prolactin plasma levels (ng/ml; *n* = 8)	41.46 (36.21)	44.24 (42.79)	*Z* = −0.7	*p* = 0.48

Abbreviations: UKU: Udvalg for Kliniske Undersogelser side effects scale.

* Significant value *p* < 0.05.

## Discussion

In this multicentric 6-month mirror-image study, we found that combining clozapine and paliperidone palmitate was an effective and safe strategy to treat patients with TRS and related psychotic disorders. After 6 months under this combination, participants showed a significant relief of symptoms, decrease in the number and length of hospitalizations, decrease in the number of visits to emergency services and increase of the functionality compared to the previous 6-month period. After an exhaustive search of side effects, this combination of antipsychotics resulted in general well-tolerated. To the best of our knowledge, this is the first study that has studied this combination in such a large sample and with this kind of mirror-image methodology.

Given that a relevant proportion of patients with a psychotic disorder do not respond adequately to monotherapy with clozapine, there is a need to know which coadjuvant treatments can be more effective and safer [4,7,8,43–46]. The combination of clozapine with LAI antipsychotics could be one of the strategies with more potential [3,47], especially with the appearance of monthly or quarterly second-generation antipsychotics.

To date, limited evidence is available about the combination of clozapine and paliperidone palmitate. A potential interest could be inferred from some short case-series which have already reported good results from adding oral paliperidone to clozapine or changing from clozapine to paliperidone palmitate [48–50]. Also, from a pharmacological point of view, the serotonin 5-HT_2A_ antagonism and dopamine D_2_ antagonism effects of paliperidone could enhance clozapine antipsychotic action, without potentiating the side effects associated with other receptors (muscarinic, histaminic, etc.). Despite the fact that patients treated with clozapine have lower treatment withdrawal rates compared to other oral antipsychotics [10,51], part of the positive results of our study could be due to the fact that an injectable formulation of paliperidone facilitates therapeutic adherence in patients with partial compliance.

Other secondary benefit detected in this study was a significant decrease in the proportion of patients with other antipsychotics and benzodiazepines, an indirect sign of the psychopathology improvement detected in the clinical scales. Although mean daily doses of clozapine decreased with the addition of paliperidone palmitate, it did not reach a significant statistical significance (from 330.47 to 265.50 mg/day; *p* = 0.062), despite its important clinical relevance. The reduction of concomitant treatments (mainly other antipsychotics and benzodiazepines, see Table 2 for details) and the decrease in clozapine daily doses could be a potential mediator in the detected decrease of number and severity of side effects, mainly psychic and neurologic (see Table 4). Although the heterogeneity of previous treatment regimens has not allowed us to do more concrete analysis, another potential reason of this findings could be related to participants switching from haloperidol, zuclopenthixol depot or even injectable risperidone to paliperidone palmitate as a coadjuvant of clozapine.

None of the potential predictors of response studied reached statistical significance. Age at index-date showed a statistical tendency (*p* = 0.066), being mean age at index-date lower for responders than for non-responders (24.1 vs. 35.75 years old). A bigger sample size of each group could have permitted to reach statistical significance, indicating that this strategy could be more useful if it is established soon.

This study has a number of limitations. First, the retrospective and naturalistic design of this study and the consequent absence of a control group is an important limitation of this observational study, as well as the moderate sample size included. Second, in a mirror-image study, each patient serves as his or her own control and observed changes from pre- to post-association introduction may reflect regression to the mean. However, this is the most used design to compare two treatments in the same individual. Third, studying last resort medications in treatment-resistant patients could generate expectation bias. Fourth, clozapine and prolactin blood levels were not available in all the participants included in this study. Fifth, the male versus female ratio of the sample included (74% vs. 26%) could be confusing some of the outcomes, such as side-effect incidence and intensity. Sixth, our results point that this treatment combination is safe on the basis of the evaluated side effects rates, but other side effects such as metabolic, that also could have a major impact on the patients’ global well-being and are associated to clozapine treatment in some subjects [52], were not assessed in this study. Besides, being a naturalistic study in real-world settings, the treatment regimen of the patients during the precombination period was heterogeneous (for instance, while 10 patients were being treated with monotherapy during that period, the rest were under diverse combination regimens). This fact did not allow to perform specific subanalyses to determine which previous treatment regimens could benefit most of changing to this treatment combination, for instance, due to the reduction of certain side effects. Finally, these results cannot be generalized to other than the mentioned LAI antipsychotics.

Despite these limitations, we believe that our study provides the first evidence that combining clozapine with paliperidone palmitate in patients with TRS and other psychotic disorders could be efficacious and safer. This augmentation strategy could help in improving the functionality and prognosis of these severe disorders, which deserve more attention in future research with randomized controlled trials of clozapine augmentation strategies for patients who failed to respond to clozapine.

## Conflicts of Interest

Dr. Amoretti reports no conflict of interest. Dr. Bernardo has been a consultant for, received grant/research support and honoraria from, and been on the speakers/advisory board of Adamed, Angelini, Casen Recordati, Janssen-Cilag, Lundbeck, Otsuka, Menarini and Takeda. Dr. Bioque has received honoraria from talks and consultancy of Adamed, has received honoraria from talks and consultancy of Angelini, has received honoraria from consultancy of Ferrer, has received research support and honoraria from talks and consultancy of Janssen-Cilag, has received honoraria from talks and consultancy of Lundbeck, has received honoraria from talks of Neuraxpharm, has received honoraria from talks and consultancy of Otsuka, and a research prize from Pfizer. Dr. Boix-Quintana has received honoraria from talks of Otsuka. Dr. Fortea has received honoraria from talks of Janssen-Cilag and Lundbeck. Dr. García-Rizo has received honoraria/travel support from Janssen-Cilag, Lundbeck and Adamed and Alter. Dr.Oriolo has received honoraria from talks of Janssen-Cilag. Dr. Palau has received honoraria/travel support from Janssen-Cilag, Lundbeck, Adamed. Dr. Parellada has received honoraria and/or research grants from the Fondo de Investigación Sanitaria of the Spanish Ministry of Science and Innovation, MINECO, Pons Balmes Grant, Fundació la Marató de TV3 of Catalonia, Janssen-Cilag, GlaxoSmithKline, Ferrer and ADAMED. Dr. Safont has received honoraria from talks of Adamed, Janssen-Cilag and Lundbeck.

## Authorship Contributions

Dr. Bioque conducted the literature review, designed the study, recruited participants, collected data, conducted the main statistical analysis, wrote the first draft of the manuscript and handled subsequent drafts after receiving coauthors feedback. Dr Bernardo, Dr. Parellada and Dr. García-Rizo designed the study, recruited participants and commented on drafts. The rest of coauthors recruited participants, collected data and commented on drafts. All of the authors contributed to the final version of the paper.

## Data Availability Statement

Due to ethical concerns, supporting data cannot be made openly available. The SPP data file used to support the findings of this study are available to bona fide researchers, subject to registration, from the corresponding author upon request.
